# Hydrogel Based 3-Dimensional (3D) System for Toxicity and High-Throughput (HTP) Analysis for Cultured Murine Ovarian Follicles

**DOI:** 10.1371/journal.pone.0140205

**Published:** 2015-10-09

**Authors:** Hong Zhou, Malika Amattullah Malik, Aarthi Arab, Matthew Thomas Hill, Ariella Shikanov

**Affiliations:** 1 Department of Biomedical Engineering, University of Michigan, Ann Arbor, Michigan, 48109, United States of America; 2 College of Literature, Science and the Arts, University of Michigan, Ann Arbor, Michigan, 48109, United States of America; 3 Department of Macromolecular Science and Engineering, University of Michigan, Ann Arbor, Michigan, 48109, United States of America; University of Missouri, UNITED STATES

## Abstract

Various toxicants, drugs and their metabolites carry potential ovarian toxicity. Ovarian follicles, the functional unit of the ovary, are susceptible to this type of damage at all stages of their development. However, despite of the large scale of potential negative impacts, assays that study ovarian toxicity are limited. Exposure of cultured ovarian follicles to toxicants of interest served as an important tool for evaluation of toxic effects for decades. Mouse follicles cultured on the bottom of a culture dish continue to serve an important approach for mechanistic studies. In this paper, we demonstrated the usefulness of a hydrogel based 3-dimensional (3D) mouse ovarian follicle culture as a tool to study ovarian toxicity in a different setup. The 3D *in vitro* culture, based on fibrin alginate interpenetrating network (FA-IPN), preserves the architecture of the ovarian follicle and physiological structure-function relationship. We applied the novel 3D high-throughput (HTP) *in vitro* ovarian follicle culture system to study the ovotoxic effects of an anti-cancer drug, Doxorobucin (DXR). The fibrin component in the system is degraded by plasmin and appears as a clear circle around the encapsulated follicle. The degradation area of the follicle is strongly correlated with follicle survival and growth. To analyze fibrin degradation in a high throughput manner, we created a custom MATLAB® code that converts brightfield micrographs of follicles encapsulated in FA-IPN to binary images, followed by image analysis. We did not observe any significant difference between manually processed images to the automated MATLAB® method, thereby confirming that the automated program is suitable to measure fibrin degradation to evaluate follicle health. The cultured follicles were treated with DXR at concentrations ranging from 0.005 nM to 200 nM, corresponding to the therapeutic plasma levels of DXR in patients. Follicles treated with DXR demonstrated decreased survival rate in greater DXR concentrations. We observed partial follicle survival of 35% ± 3% (n = 80) in 0.01nM treatment and 48% ± 2% (n = 92) in 0.005nM, which we identified as the IC50 for secondary follicles. In summary, we established a 3D *in vitro* ovarian follicle culture system that could be used in an HTP approach to measure toxic effects on ovarian follicles.

## Introduction

In the US, large quantities of pharmaceuticals are produced and introduced into commerce annually [1]. Evaluation and prediction of potential ovarian toxicity is therefore a public health priority. Despite the myriad compounds people are exposed to in daily life, however, there is no standard rapid and efficient screening for the potential reproductive health effects of these compounds. Current guidelines for reproductive toxicity testing set forth by the International Conference on Harmonization of Technical Requirement for Registration of Pharmaceuticals for Human Use, as well as the Guidelines for Reproductive Toxicity Risk Assignment issued by the US Environmental Protection Agency, rely primarily on *in vivo* animal studies. Based on a recent report from EPA, a complete set of *in vivo* tests for a single chemical compound requires use of thousands of animals, making the cost and time spent prohibitive [2]. This difficulty has motivated the development of *in vitro* high-throughput (HTP) screening assays to identify high-priority chemicals for in-depth investigation.

Ovarian follicles are the functional and morphological units composed of a germ cell (the oocyte) and multiple surrounding somatic cells (theca and granulosa cells). The somatic cells produce hormones necessary for the regulation of the reproductive system, and support the oocyte in its maintenance, growth and development. During the process of follicular growth, i.e., folliculogenesis, the cross-talk between the germ and somatic compartments enables the enclosed immature oocyte to develop into a mature fertilizable ovum [3]. Xenobiotic exposure during this process can lead to deleterious effects on folliculogenesis, resulting in oocyte incompetence, genetic and epigenetic changes, and misregulated hormone production. As such, it affects the reproductive system at different levels [4,5]. Hence, *in vitro* follicle growth can serve as a simple, rapid and robust tool to screen the effects of compounds on female reproductive function [6]. A significant amount of ovarian toxicity data has been obtained using the mouse *in vitro* follicle growth, which supports mouse ovarian follicle growth, development, and maturation of intact early pre-antral follicles over a culture period of 10–13 days with continuous toxicant exposure [7–14].

However, *in vitro* cell-based assays and subsequent preclinical *in vivo* studies may not yet provide sufficient pharmacological and toxicity data, as evidenced by substantial percentages of new chemical and biological entities that fail in human clinical studies or are removed from the market for safety reasons [15]. A recent review compared different assays for toxicity assessment and highlighted the importance of 3D scaffolds that resemble native microenvironment, supply appropriate topology, and provide mechanical stimulation similar to that present *in vivo* [16]. Hence, addressing such questions remains critical to asserting the validity of *in vitro* cell-based test system in toxicology in order to confidently and adequately elucidate important physiological issues [17]. For example, one of the important components of follicular microenvironments is the antrum. It is a closed, fluid-filled cavity, which forms around the oocyte as a follicle expands. It is also considered a milestone in follicular development. Residing in the center of avascular growing follicles, the expansion of the antrum and the formation of the follicular fluid within is a key indicator of follicular growth, heralding the development of the follicle to pre-ovulatory stage. Formation and maintenance of a fluid filled enclosed cavity was demonstrated in a fibrin-alginate hydrogel based 3D follicle culture [18]. Molecules in the follicular fluid include hyaluronan, chondroitin sulfate proteoglycan versican and inter-α-trypsin inhibitor, suggesting that the antrum is osmotically active [19,20]. In addition, the presence of antrum may serve as a buffering system, protecting follicles from abrupt changes in hormone concentrations or toxic exposure.

In this study we aimed to design a hydrogel-based 3D report system that allows continuous morphological, genetic and biochemical sampling of ovarian follicle cultured in *in-vivo*-like conditions. Fibrin-alginate interpenetrating network (FA-IPN) [18,21] is a dynamic culture system for *in vitro* follicle cultures. Naturally derived, alginate is slowly degrading and biologically inert to ovarian tissue. As such, it provides structural support for the encapsulated follicles for an extended period of culture lasting 10 to 12 days. Fibrin however, is a biologically active protein and degrades as follicles grow and secrete proteolytic enzymes in response to follicle stimulating hormone (FSH). Fibrin degradation appears as a clear growing circle around the encapsulated follicle as follicles grow in the culture. Importantly, if the follicle is damaged, fibrin degradation significantly slows down or stops due to lack of the proteolytic activity normally carried out by proteases secreted from functioning granulosa cells of a viable follicle [18,21]. As a result, follicle health and survival can be correlated with the proteolytic activity by quantifying optical clearance due to fibrin degradation, which serves as the base for the proposed system.

Following the initial design stage, we validated the system performance by exposing cultured follicles to doxorubicin (DXR), a moderately ovotoxic chemotherapeutic agent [22–24]. Follicles exposed to increasing doses of DXR had a decreasing survival rate, coupled with attenuated fibrin degradation. We utilized MATLAB®’s image processing toolbox to capture the changes in follicle dimensions and degradation of surrounding matrix. This approach allowed quantitative analysis of the effect of toxicants on folliculogenesis and comparison of multiple conditions with variations in duration of exposure, toxicant types and/or concentration.

## Material and Methods

All chemicals were purchased from Sigma-Aldrich (St. Louis, MO) unless otherwise specified. Media formulations were purchased from Invitrogen (Carlsbad, CA). Nalgin MV-120 sodium alginate (Carrageenan, Xanthan, Alginate, Ingredients Solution, Inc., Waldo, ME, USA) and the fibrinogen-thrombin kits (Baxter Healthcare, BioScience Division, Westlake Village, CA) were used for fibrin-alginate gel formation.

### 1. Animals

Mice were purchased (Harlan, Indianapolis, IN), and housed in ventilated cages in a temperature and light controlled environment (12L: 12D) and provided with food and water. Animals were treated in accordance with the guidelines and regulations set forth by the National Institutes of Health Guide for the Care and Use of Laboratory Animals and the established Institutional Animal Use and Care protocol at the University of Michigan. Experiments with animals were performed in strict accordance with the protocols approved by the Institutional Animal Care and Use Committee (IACUC) at the University of Michigan (PRO00004106). All animals used in this study were euthanized by overdose of isoflurane via inhalation followed by decapitation.

### 2. Follicle isolation, encapsulation and culture

Two-layered secondary follicles with diameters ranging from 120 to 135 μm were mechanically isolated from ovaries of 12 to 14 days old female F1 hybrids (C57BL/6JRccHsd inbred × CBA/J CrHsd). Individual follicles were encapsulated in droplets of 7.5 μL fibrin alginate gel based on “the drop method” described by Shikanov et al [21]. Briefly, the final fibrin alginate mixture consisted of 12.5 mg/mL fibrinogen and 0.2% (w/v) alginate while thrombin concentration was adjusted to 12.5 mg/mL. The cross-linking time for beads in thrombin/Ca^2+^ solution was 2 minutes. Encapsulated follicles were cultured in α-MEM based growth media (GM), supplemented with 1 mg/mL Fetuin, 3 mg/mL bovine serum albumin (BSA, MP Biomedicals, Irvine, CA), 0.1% insulin-transferrin-selenium (ITS), and 10 mIU/mL recombinant human follicle stimulating hormone (rhFSH, GONAL-f ® 75 IU, EMD Serono, Rocklan, MA). Every 2 days, the cultured follicles were imaged by a light microscope (DMI 3000, Leica, Germany) with follicle diameters measured with the Leica software LAS V4.3 (Leica, Germany) while half (50 μL) of the growth media was replaced by pre-equilibrated, fresh media.

To control fibrin degradation, we used plasmin inhibitor, aprotinin, at concentrations ranging from 0.025 to 0.05 TIU/mL. A stock solution of aprotinin (Apr) was dissolved in DPBS and then diluted with growth media before media changing.

### 3. *In vitro* follicle maturation (IVFM) and oocyte collection

After 12 days of culture, healthy-looking follicles with diameters ranging between 300 to 350 μm were identified. The alginate was degraded by placing the FA beads with the follicles in 100 μL αMEM medium containing 1% fetal bovine serum (FBS) and 10 mIU/mL alginate lyase (MP Biomedicals, Irvine, CA) for 2 minutes at 37°C and 5% CO_2_. After retrieving the follicles from the beads, whole follicles were transferred to an IVF dish with maturation media composed of αMEM, 10% FBS, 5 ng/mL epithelial growth factor (EGF), 1.5 IU/mL human chorionic gonadotropin (hCG), and 10 mIU/ml rhFSH for 12–14 hours at 37°C and 5% CO_2_. Cumulus-oocyte complexes (COCs) underwent cumulus expansion, and the oocytes from the expanded COCs were collected and isolated from the surrounding cumulus granulosa cells by adding 0.1% hyaluronidase and gentle aspiration with a pipette. The oocytes were considered at metaphase II (MII) if a polar body was present in the perivitelline space.

### 4. Quantitative Measurement

We used ImageJ (Rasband, W.S., ImageJ, National Institutes of Health, Bethesda, MD, USA, http://imagej.nih.gov/ij/, 1997–2014) to process the images for the manual and automated quantitative measurements. For the manual calculation, areas of fibrin degradation were measured using the “Straight” tool in ImageJ by drawing two orthogonal lines across the degraded area. We then calculated the area (A) of fibrin degradation using the diameter results ($A\;=\;\pi {\left(\frac{d}{2}\right)}^{2}$). For the automated analysis, the brightfield color images were converted to binary images using our custom MATLAB® program (S1 File and S2 File). To achieve this, a threshold value was first determined by the program automatically analyzing the micrographs with evenly exposed background. Then, areas with values higher than the defined threshold were converted to black pixels, correlating with the non-degraded fibrin, whereas areas with lower than threshold values were converted to white pixels, correlating with the degraded fibrin the binary images. Before submitting images for program to analyze fibrin degradation area, the sizes of follicles were calculated to compensate the area loss in the fibrin degradation circle due to follicle expansion using ImageJ. Then, area of degraded fibrin was measured by using the “Analyze” menu. Fibrin degradation area from both methods (M for manual calculation and A for program measured areas) were recorded and compared as a factor indicating follicle health.

### 5. Exposure of secondary follicles to doxorubicin (DXR)

We added doxorubicin (DXR) to the cultured follicles on D0, D2 and D4 of the culture at the concentrations ranging from 0.005 nM to 200 nM. For all DXR treatments in this study, a stock solution of DXR was dissolved in dimethylsulfoxide (DMSO) and then serially diluted in growth media. The final concentration of DMSO was kept at 0.05% at all DXR conditions, which is consistent with previous publications [11]. Doxorubicin actual concentrations in growth media were confirmed by measuring the auto-fluorescence of DXR using a fluorescence plate reader at room temperature (Fluoroskan Ascent, Labsystems, Finland, excitation 490 nm, emission 590nm). The calibration curve for DXR concentrations from 10 nM to 400 nM had a R^2^ value of 0.99. Solutions of DXR below the detection limit of the plate reader were achieved by sequential dilutions of the 10 nM solution.

### 6. Statistical analysis

All statistical analyses were performed using GraphPad Prism (GraphPad Prism Software, La Jolla, CA). Two-sided t-test was performed to determine differences in follicle diameters between control and aprotinin treatment group at any given time point and fibrin degradation area data between the two groups. One-sided t-test was adapted by the MATLAB® to output follicle health evaluation based on fibrin degradation calculation. Values of p < 0.05 were considered statistically significant.

## Results and Discussion

### 1. Optimal aprotinin concentration preserved intact fibrin degradation circle without affecting follicle health

During mouse ovarian follicle culture, the fibrin component in the FA-IPN is gradually degraded and the products of fibrin proteolysis diffuse through the hydrogel. The initial clearance occurs in the area adjacent to the follicle and then radially progressing outwards to the edge of the hydrogel bead [18]. The fibrin removal mirrors the follicle growth and volumetric expansion whereas the much slower degrading alginate remains as a structural and mechanical support. Thus, it is important to have both fibrin and alginate in the IPN to support folliculogenesis *in vitro* and for the fibrin component to serve as an optical density indicator for the follicle growth. Healthy growing follicles demonstrate strong proteolytic activity, which results in fast fibrin degradation. Addition of aprotinin, a small molecule that inhibits plasmin proteolytic degradation, resulted in slower fibrin degradation and more controllable optical density change, which lasted over an extended period of time.

**Fig 1:**
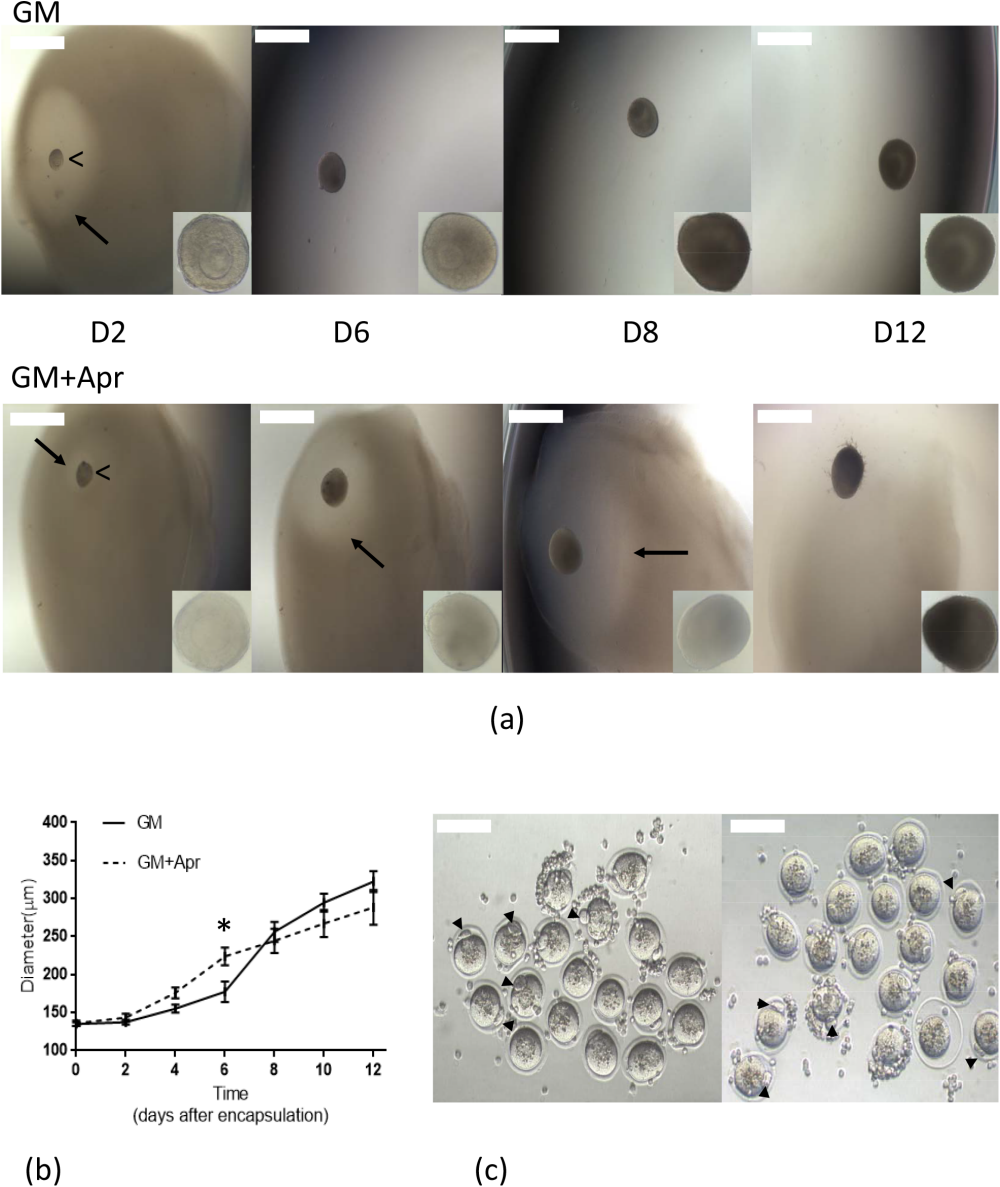
Aprotinin-controlled fibrin degradation. (a) Representative images of follicles encapsulated in FA-IPNs on different days of culture with higher magnification images of follicles in the lower right inserts. Comparison showed that optimized aprotinin concentration (0.025 to 0.05 TIU/mL) effectively delayed fibrin degradation till D8, a critical time for antrum formation. Arrow points to the fibrin degradation area surrounding the growing follicle. Arrow head (“<”) points to the encapsulated follicle in the FA-IPN. Scale bar = 500 μm. (b) Growth curves of follicles cultured in growth media with and without aprotinin. Data are shown as mean ± SEM, p < 0.05. n (total follicles, GM) = 286, n (total follicles, GM+Apr) = 258, n (total animals used) = 30, n (experiments) = 10 for each condition. (c) Representative images of *in vitro* follicle maturation (IVFM) results confirming oocyte quality from both GM (left) and GM+Apr (right) conditions. Data are shown as mean ± SEM, p < 0.05. MII rate (GM) = 78% ± 3%, MII rate (GM+Apr) = 81% ± 3%. Scale bar = 100 μm.

We have optimized the concentration of aprotinin in the culture media to delay the degradation of fibrin up to 8 days after encapsulation (D8). Our results showed that aprotinin added to the media in the range between 0.025–0.05 TIU/mL resulted in controlled fibrin degradation and antrum formation on day 8 in cultured follicles (Fig 1A). Suboptimal concentrations of aprotinin (less than 0.01 TIU/mL) resulted in fast fibrin degradation, and concentrations above 0.1 TIU/mL led to follicle death due to matrix stiffness [21].

We examined the appearance of the cultured follicles at magnification of 20X to further distinguish between surviving and dead follicles. Follicle survival rate was 73% ± 3% without aprotinin and 70% ± 3% with aprotinin (Table 1), which was not statistically significant. Follicle diameters in both conditions, with and without aprotinin, reached 322±12 μm and 288±23 μm respectively after 12 days of culture (Fig 1B). To confirm follicle health, we compared the rate of oocyte maturation of the oocytes harvested from follicles cultured in both conditions by *in vitro* follicle maturation (IVFM) (Fig 1C). Follicle health and oocyte maturation rate was not affected by the aprotinin treatment and we did not observe significant differences in survival rate or metaphase II (MII) rate (78% ± 3% VS 81% ± 3%, Table 1) between the two conditions.

**Table 1:** Summary of follicle growth and oocyte maturation under different conditions.

	%survival†	%antrum formation	Diameter (D6, μm)	Diameter (D12, μm)	%MII†	%GV†	%MI†	%DG†
GM	73±3^a^	87±2 ^a^	177±5 ^a^	322±12 ^a^	78±3 ^a^	6±0 ^a^	12±1 ^a^	4±1 ^a^
GM+Apr	70±3^a^	86±2 ^a^	224±12 ^b^	288±23 ^a^	81±3 ^a^	5±0 ^a^	10±1 ^a^	4±1 ^a^
DXR treatments								
200 nM	0	0	-	-	-	-	-	-
10 nM	0	0	-	-	-	-	-	-
1 nM	0	0	-	-	-	-	-	-
0.1 nM	0	0	-	-	-	-	-	-
0.01 nM(survived)	35±3^b^	27±3 ^b^	252±18 ^a^	307±15 ^a^	-	29±1 ^b^	-	71±2 ^b^
0.005 nM (survived))	48±2 ^c^	64±3 ^c^	251±19 ^a^	327±14 ^a^	-	-	70±2 ^b^	30±2 ^c^

†: %survival = (the number of survived follicles) ÷ (total number of follicles cultured)×100

%antrum = (the number of follicles with antrum) ÷ (the number of survived follicles)×100; %MII = (the number of MII oocytes) ÷ (total number of follicles matured) ×100; %GV = (the number of GV oocytes) ÷ (total number of follicles matured) ×100; %MI = (the number of MI oocytes) ÷ (total number of follicles matured) ×100; %DG = (the number of MII oocytes) ÷ (total number of follicles matured) ×100. Follicle diameter data was presented as mean ± SEM. Different letters (a, b, and c) within each column indicate statistical significance (p<0.05).

### 2. *In vitro* fibrin degradation positively correlated with *in vitro* folliculogenesis, serving as the foundation of the proposed system

**Fig 2:**
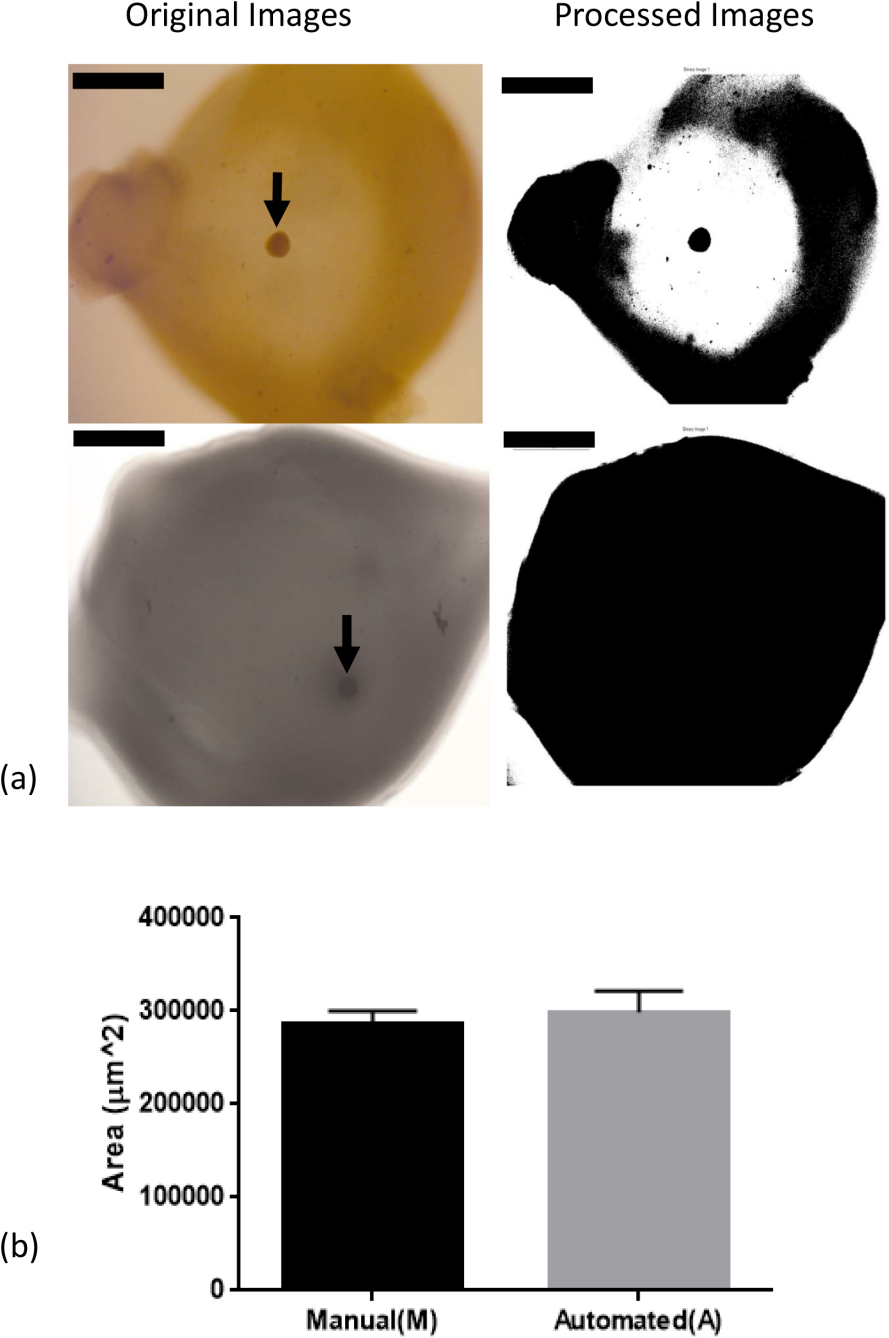
Correlation of fibrin degradation and follicle health, evaluated by both manual calculation (M) and automated MATLAB ® program (A). (a) Representative images of a live follicle (top) and a dead follicle (bottom) in brightfield images and processed binary images. Scale bar = 800 μm. (b) Results from manual calculation (M) and automated program measurement (A) of fibrin degradation in GM+Apr condition for live follicles on D6. Data presented as mean ± SEM, with n = 9 in each case.

The FA-IPN has been shown to promote mouse follicle growth, increasing the number of meiotically competent oocytes relative to either fibrin or alginate alone [18]. In mice, ovarian follicles secrete plasminogen activator in response to FSH stimulation and degrade fibrin in the encapsulating hydrogel matrix. We therefore established a correlation between fibrin degradation and follicle health, demonstrating that healthy follicles can actively degrade fibrin in the FA-IPN during culture, while damaged ones cannot. Examples of originally captured images and images processed using the MATLAB® code are shown in Fig 2A. The live follicle (arrow, left column, top) degraded the fibrin component during 6 days of culture and was surrounded by clear area, while the matrix around the dead follicle (arrow, left column, bottom) remained dark and dense. We specifically chose D6 images for this quantification because of the correlation between antrum formation and follicle atresia [19,20]. These images were processed with the MATLAB® code and the right column in Fig 2A shows the processed binary images in which the fibrin degradation circles were intensified by this conversion. We used both manual and automated MATLAB®/ImageJ [25] to measure changes in optical clarity due to fibrin removal and the results from both manual calculation and program measurements were recorded and compared in Fig 2B. We did not see any significant difference between the two methods, confirming that the program is suitable to measure fibrin degradation in an attempt to evaluate follicle health. However, a slightly greater variation was observed in program measurements, which could be due to the more forgiving aspect of the manual calculation.

**Fig 3:**
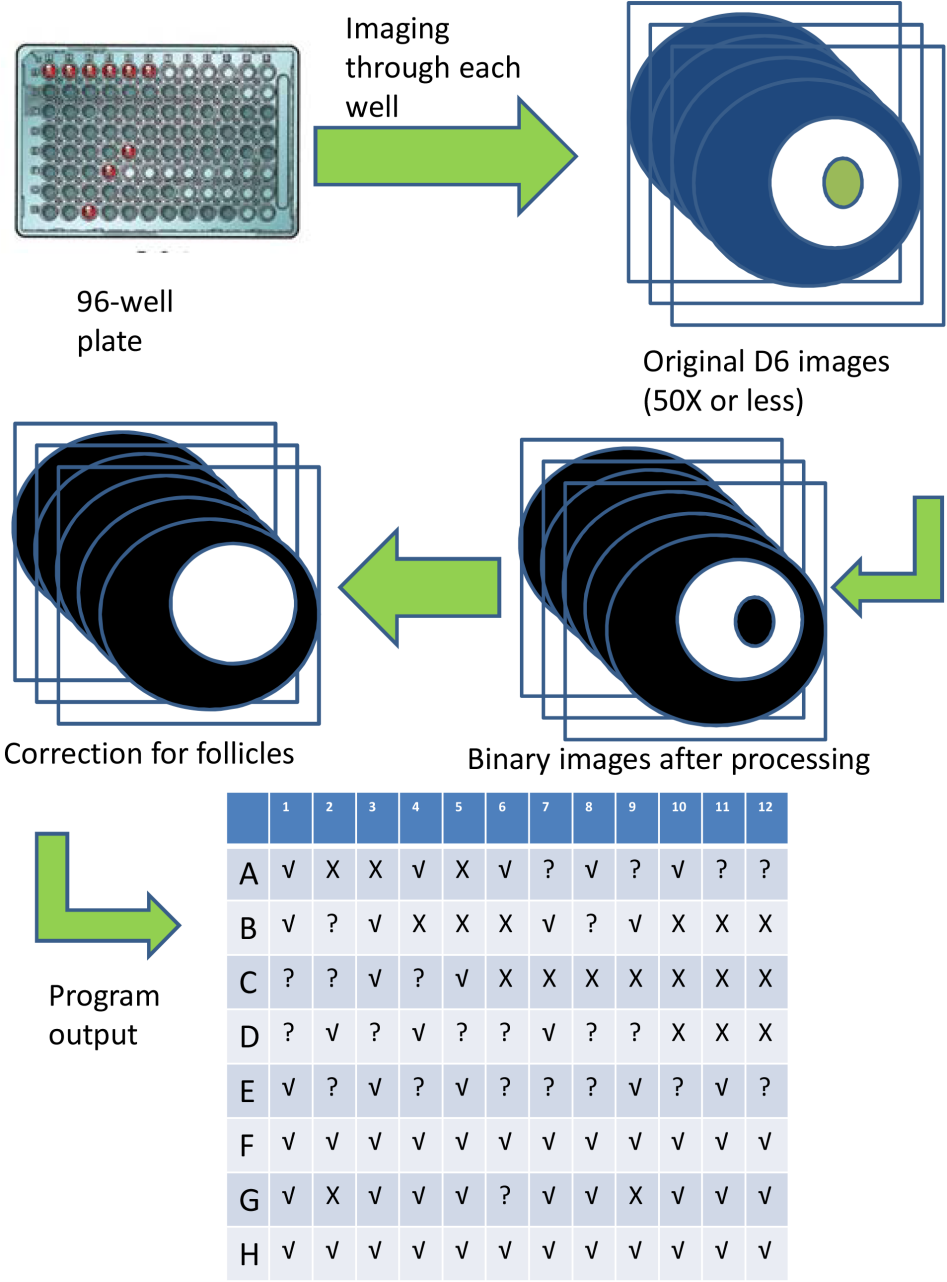
Illustration of the HTP system. Images captured from 96-well plates are converted using MATLAB ® program to binary images. After comparing the area of fibrin degradation (shown as white circles) to control conditions, the program will then output results of follicle health evaluated by fibrin degradation: “√” indicates healthy follicles, “X” dead ones, and “?” requiring further evaluation.

The proposed screening process for the high-throughput approach is demonstrated in the flow chart of the image analysis in Fig 3. Stacks of original images of FA-IPNs are first converted to binary images, in which original optical clarity due to fibrin removal would appear as white whereas follicles and areas with fibrin presence would appear as black. Since the “black” area that correlates with the encapsulated follicles reside in the white area of fibrin degradation, we can correct for area loss due to follicle expansion for further MATLAB® program analyses. The program output is based on the fibrin degradation area measurement of the current images to the cutting-off value [mean– 1.5× standard deviation (SD)] established by the controls and normal distribution assumption. The system was designed to test for positive toxic effects on follicle health, therefore if the fibrin degradation area is significantly smaller (< cutting-off value), the output will be “X”, indicating positive toxic effects, i.e., dead follicles; if the degradation area is comparable to controls (> cutting-off value), the output is “√”, indicating absence of toxic effects, i.e., healthy follicles; if the degradation area is equal to the cutting-off value, the p-value in this case is equal to 0.05, which may require further examination at higher magnification.

The position of the encapsulated follicle in the bead may also affect the measurements of fibrin degradation area. In cases where a follicle is encapsulated close to the edge of the bead and the resulted degradation area is limited by the edge, the MATLAB program still compares the radii of 2 follicle-centered circles: the radius of actual fibrin degradation as appeared in the image. Since part of the actual fibrin degradation area is “missing” (degradation reaches the edge), the program will output a “?” instead of “√”, which indicates the follicle needs further evaluation by the experimenter such as manually comparing the radius and examining the follicle with higher magnification under the microscope.

### 3. Doxorubicin treatments confirmed system functionality and identified the IC50 for secondary follicles

To test our system, we treated cultured follicles with doxorubicin (DXR), a chemotherapeutic agent used to treat multiple cancers, including breast and ovarian cancer. DXR is an anthracycline that acts by intercalating DNA, thus preventing replication and transcription. Depending on the cell type and drug dose, DXR induces breaks in topoisomerase II-dependent double-strand DNA breaks and oxidative stress [22,23]. Recent evidence suggests that doxorubicin is moderately ovotoxic via direct apoptotic effects on follicles/oocytes and/or an impact on the entire ovary, which may potentially lead to infertility and premature ovarian failure (POF) [23].

**Fig 4:**
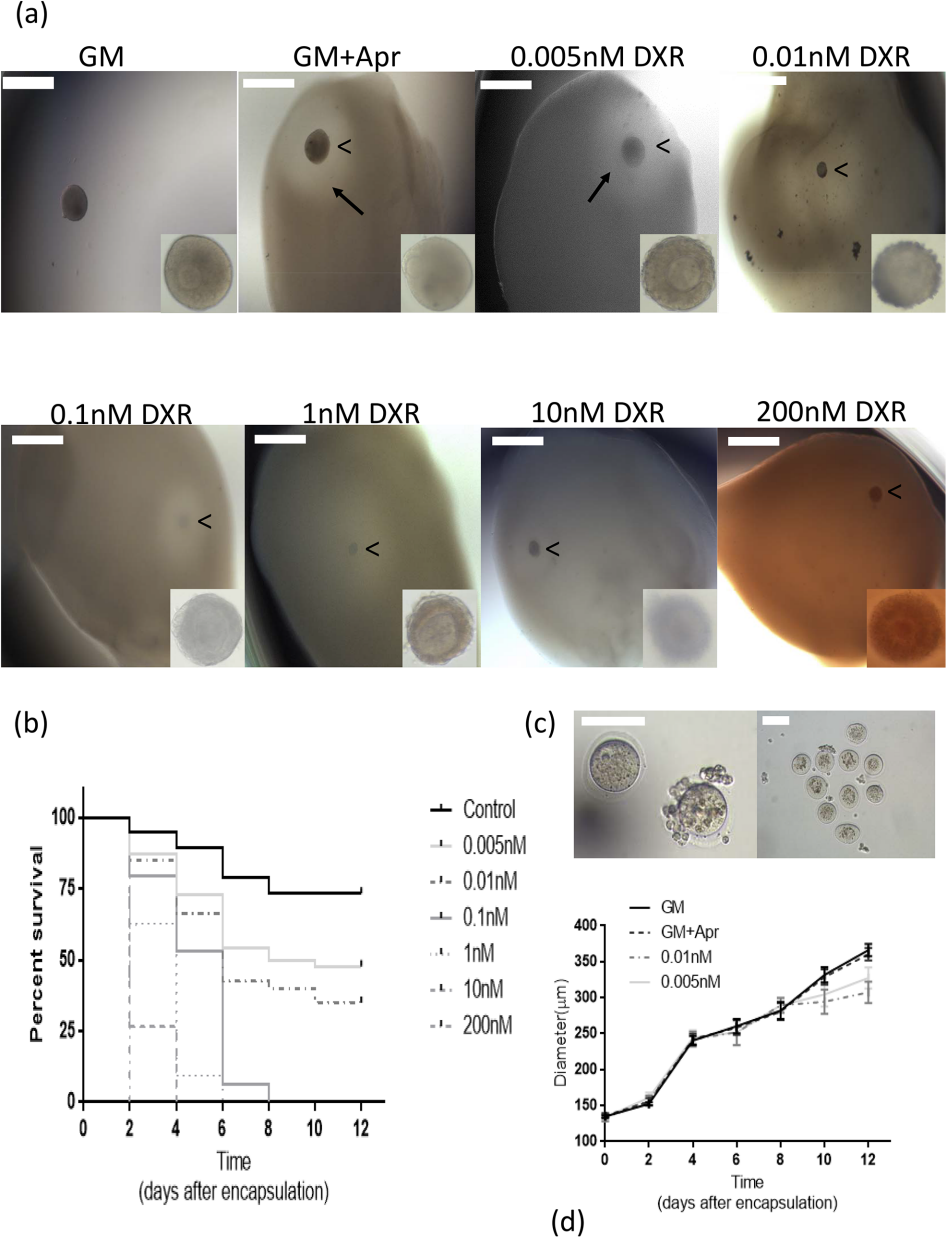
System validation with Doxorubicin (DXR). (a) Representative images of dose response of cultured follicles to DXR. Arrow points to the fibrin degradation area surrounding the growing follicle. Arrow head (“<”) points to the encapsulated follicle in the FA-IPN. Scale bar = 500 μm. (b) Kaplan-Meier cumulative survival curves for DXR treatments and control. Sample sizes for each condition [n (condition) = total follicles, animal used, experiments]: n (200 nM) = 69, 4, 3; n (10 nM) = 52, 3, 3; n (1 nM) = 75, 3, 3; n (0.1 nM) = 75, 3, 3; n (0.01 nM) = 80, 3, 3; n (0.005 nM) = 92, 4, 3. (c) Representative images of *in vitro* follicle maturation (IVFM) results from survived follicles in the 0.01 nM (left) and 0.005 nM (right) DXR treatment group. Scale bar = 100 μm. (d) The diameters of surviving follicles in control conditions (GM and GM+Apr) and 0.005 nM and 0.01 nM DXR treatment groups. Data presented as mean ± SEM.

**Fig 5:**
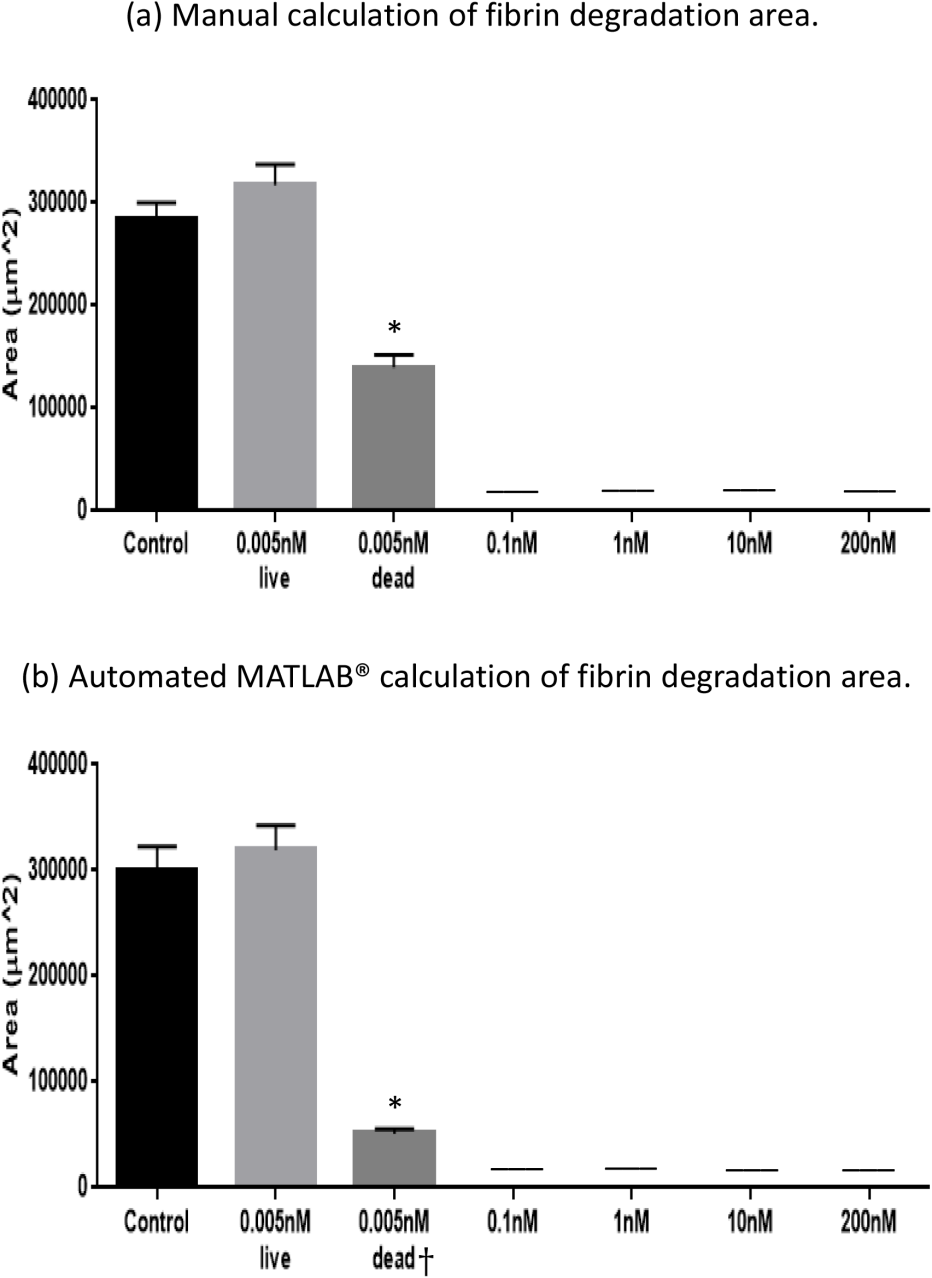
Quantitative measurements of fibrin degradation with different DXR treatments. (a) Manual calculation of fibrin degradation area; (b) Automated MATLAB® measurement of fibrin degradation. Data presented as mean ± SEM, n = 9, † n (GM+Apr+0.005nM DXR, dead, automated) = 3, *: p<0.05.

We exposed cultured mouse ovarian follicles to multiple concentrations of DXR, ranging from 0 to 200 nM (Fig 4A). This range was determined based on what have been previously established as therapeutic plasma levels in patients undergoing DXR chemotherapy [26]. None of the follicles survived in conditions with doxorubicin concentrations greater than 0.01nM (Fig 4B and 4D). Similarly, we performed a manual calculation and MATLAB® program measurement to quantify fibrin degradation by follicles exposed to different DXR concentrations (Fig 5). The degradation area around a healthy follicle cultured for 6 days reached 3×10^5^ μm^2^ and this value was set as a control. Follicles that survived the exposure to 0.005nM DXR degraded the fibrin to the same extend as the control, while follicles exposed to 0.01nM DXR had a significantly smaller area of degradation. Consistent with no follicle survival in DXR concentrations higher than 0.01 nM, there was no detectable fibrin degradation by the manual analysis and the program.

The design of our system allowed us to easily identify an absence of toxic effects (output “√”) as well as strong positive toxic effects resulting in irreversible damage to the follicle health and death (output “X”). Remarkably, it was the lower end concentrations (0.005 nM or 0.01 nM) of the DXR (output “?”) that required further analysis. Growing follicles in the “?” group showed comparable diameter increase from their initial size and the fibrin degradation area on D6 was comparable to those in control conditions. However, 70% of the oocytes retrieved from the follicles cultured in 0.005nM DXR after in vitro follicle maturation (IVFM) were arrested at MI stage and were not able to complete meiosis (Fig 4C, right). The remaining 30% of the oocytes from this group were degenerated (Table 1). Noticeably, in the treatment group with 0.01 nM DXR, the maturation of the surviving follicles resulted in 29% oocytes arrested at GV state, while the other 71% were degenerated (Fig 4C, left). The images of D8 of the growing follicles with degenerated oocytes in 0.01nM DXR treatment showed dark and dense phenotypes when examined under high magnification, despite of the increase in the follicle diameters and comparable fibrin degradation area. This morphology is a sign of unhealthy follicle development, which may indicate premature luteinization and/or apoptosis of granulosa cells and the oocyte.

The negative effect on meiosis progression could be due to the mechanism of doxorubicin. DXR acts by intercalating DNA, and therefore prevents replication and transcription. DXR interferes with the actively proliferating granulosa cells during folliculogenesis, leading to impaired granulosa cell functions. As previously reported, DXR first accumulates in ovarian stromal cells, and then progressively shifts radially inwards to penetrate follicles and reach sufficient nuclear concentrations in granulosa cells [22]. This temporal insult pattern in granulosa cells could contribute to the initial diameter increase and fibrin degradation we observed in the beginning of culture with lower concentrations of DXR. The slower timeline for granulosa cell damage is suggestive of a window to prevent acute DXR ovarian insult and follicle demise as recently reported [27]. A treatment with Bortezomib, a DXR competitive binder to the proteasome and translocation to the DNA, prior to DXR exposure attenuated DXR-induced DNA damage in all ovarian cell types, resulting in apoptosis of preantral follicles *in vivo* [27]. These findings support our observations of continued follicle growth during culture but impaired oocyte maturation on D12.

In this study, we tested a single toxicant, DXR, for its established ovotoxicity and relatively linear dose-dependent responses. As our results indicated, we established a direct correlation between the lack of follicle growth and ovotoxicity using DXR. This correlation has the translational potential to study the effects of other toxicants. Mouse secondary ovarian follicles encapsulated in FA-IPN grow and degrade fibrin, or die/arrest at early stages followed by a halt of fibrin degradation, which allowed us to make a definitive conclusion regarding the agent’s toxicity. Importantly, human and nonhuman primate follicles cultured in hydrogel based 3D systems [28–30] presented 3 different phenotypes of “no-grow”, “slow-grow”, and “fast-grow” cohorts, which complicates the application of this approach for toxicity screening in higher species’ follicles. Thus further biomaterial design and advanced image processing tools for our system is required to be able to predict ovarian toxicity in human and primate follicles.

## Conclusion

We developed a program-based analysis of fibrin matrix degradation around a cultured follicle as a reporting tool for follicle health. We used mouse ovarian follicles cultured in vitro as a tool to screen and prioritize ovotoxicants in a physiologically relevant environment. The designed hydrogel based 3D HTP in vitro ovarian follicle culture system using FA-IPN was successfully tested to accurately report the ovotoxic effects of Doxorubicin. The control of the fibrin degradation rate allowed toxicity screening for extended periods of culture, without disruption of follicle development. The customized automated MATLAB® image processing software supported the hands-off approach to screen multiple conditions in a high throughput manner. Mechanistic studies utilizing this approach have the potential to evaluate synergistic and additive effects of toxicants’ mixtures.

## Supporting Information

S1 File MATLAB® code for binary image conversion. (DOCX)

S2 File MATLAB® code for fibrin degradation area comparison. (DOCX)
