# Supplementary material for: Hydrogel Based 3-Dimensional (3D) System for Toxicity and High-Throughput (HTP) Analysis for Cultured Murine Ovarian Follicles
Source: PLoS One. 2015 Oct 9;10(10):e0140205. doi: 10.1371/journal.pone.0140205 (PMC4599796; doi:10.1371/journal.pone.0140205)
Supplement: S1 File — (DOCX) [file pone.0140205.s001.docx]

function ImageProcessor

clear all

condition = 0;

while condition == 0

prompt = 'Please input whether the image was taken using a 5x, 10x, or 20x scope by entering either 5, 10, or 20: ';

type = input(prompt);

if type == 5

conversion = 1.0597; %Conversion for 5X is 1.0597 pixels/micron^2

condition = 1;

elseif type == 10

conversion = 4.16159; %Conversion for 10X is 4.16159 pixels/micron^2

condition = 1;

elseif type == 20

conversion = 9; %Converstion for 20x is 9.0 pixels/micron^2

condition = 1;

else

condition = 0;

disp('The image size was not valid, please enter 5, 10, or 20 to denote the image size');

end

end

prompt2 = 'Please input the image name using the appropriate extension: ';

name = input(prompt2,'s');

%Reading in the image

image1 = imread(name);

figure

imshow(image1);

title('Initial Image');

map = colormap;

%Adjust Contrast: Step 1

%Convert to LAB and adapt and equalize the histogram of the luminosity

%channel

image2 = image1;

srgb2lab = makecform('srgb2lab');

lab2srgb = makecform('lab2srgb');

image1lab = applycform(image1,srgb2lab);

labimage1 = applycform(image1,srgb2lab);

labimage1(:,:,1) = adapthisteq(labimage1(:,:,1));

labimage1(:,:,1) = histeq(labimage1(:,:,1));

%Option of filtering to blur an image after stretching out

%labimage1(:,:,1) = medfilt2(labimage1(:,:,1),[3 3]);

%Convert back to RGB and display results of first contrast adjustment

im1 = applycform(labimage1,lab2srgb);

figure

imshow(im1);

title('Im1');

%Adjust Contrast: Step 2

%Stretch out histogram of colors for greater separation, more distinct

%light and dark channels

stretch = imadjust(im1,stretchlim(im1),[]);

figure

imshow(stretch);

title('Stretchy');

%{

reverse = imcomplement(stretch);

figure

imshow(reverse);

title('Reverse');

%}

%Convert image to grayscale form, optional sharpening of image to help

%detect edges

gray = rgb2gray(stretch);

%gray = imsharpen(gray);

figure

imshow(gray);

title('Gray Image');

%{

filter = medfilt2(gray(:,:,1),[5 5]);

figure

imshow(filter);

title('Filter Gray');

%}

%Convert image to binary form, threshold determines how "white" the gray

%intensity image needs to be to show up as a white pixel on the binary

%form. Higher thresholds will require stronger white inensities to be a

%white pixel in the binary form

threshold = 0.6;

binary = im2bw(gray,threshold);

figure

imshow(binary);

title('Binary');

%Morphological Operations:

%Opening and closing the image with a small structuring element in order to

%reduce noise in the image, clarify regions of interest

%{

nose = strel('disk',1);

open = imopen(binary,nose);

figure

imshow(open);

title('Opened');

%}

nose = strel('disk',3);

close = imclose(binary,nose);

figure

imshow(close);

title('Closed');

%Taking complementary (negative image) of the binary form. All black pixels

%are changed to white and vice versa. Follicle will change from black to

%white for analysis

complement = imcomplement(close);

figure

imshow(complement);

title('Complement Closed Image');

%Drawing boundaries around regions of interest

[B,L,N] = bwboundaries(complement);

hold on

for k=1:length(B)

boundary = B{k};

if(k > N)

plot(boundary(:,2), boundary(:,1),'g','LineWidth',2);

else

plot(boundary(:,2), boundary(:,1),'r','LineWidth',2);

end

end

hold off

%Labeling all regions with random colors for analysis

[labels, number] = bwlabel(complement);

colorlabel = label2rgb(labels,'hsv','k','shuffle');

figure

imshow(colorlabel);

title('Labels');

%Measuring properties of all the colored regions. If you want to measure a

%black region, simply take the complementary image

props = regionprops(labels,complement,'all');

area = 0;

%Specifying the follicle as the region of interest for analysis and looking

%glass

follicle = 0;

%Mean diameter of follicles mutiplied by pixel conversion factor

folmean = 223.91 * conversion;

%Standard deviation of follicle diameter multiplied by pixel conversion

%factor

folstdev = 57.32 * conversion;

%Standard deviation multiplier for upper and lower bounds

stdevmult = 1.5;

upperlim = (pi)*((folmean/2) + ((stdevmult * folstdev)/2))^2;

lowerlim = (pi)*((folmean/2) - ((stdevmult * folstdev)/2))^2;

%Delete comment to see the values for the upper and lower limits

%upperlim

%lowerlim

for i = 1:number

if props(i).Area >= lowerlim && props(i).Area <= upperlim

%Looking within one standard deviation of the mean diameter of live

%follicles on day 6 for 5X images so far

%Issue: Not all follicle images have a very similar sized follicle

%depending on the conditions, may need to specify conditions

%appropriately

perimeter = props(i).Perimeter;

area = props(i).Area;

areaDetect = 1;

%Now we are also going to check if the measured perimeter of the

%object is close to what the circular perimeter estimate. This

%helps us to filter out regions of similar area that are non

%circular

circradius = sqrt(area/pi);

circperim = 2*pi*circradius;

if perimeter >= (circperim-1000) && perimeter <= (circperim + 1000)

follicle = 1;

area = props(i).Area;

center = props(i).Centroid;

folliclearea = area;

diameter = sqrt(4*area/pi);

folliclediameter = diameter;

labelnum = i;

end

end

end

if follicle == 1

hold on

plot(center(1),center(2),'Color','k','Marker','*');

hold off

disp('The estimated follicle diameter is: ')

folliclediameter/conversion

%To do: Look at the fact that dead follicles are not really detected with

%the program, relies on contrast between the follicle and degradation area

%to detect

figure

imshow(labels == labelnum);

title('Follicle Pulled Out');

%Now let's pull out a circle outside of our follicle and use

%that for an image

[col row] = meshgrid(1:2560, 1:1920);

%For now, the radius has been set arbitrarily to 300 pixels, but you

%can adjust this value according to the degradation area measurements

radius = 300;

circle = (row - center(2)).^2 + (col - center(1)).^2 <= radius.^2;

circle = mat2gray(circle);

figure

imshow(circle);

title('Expanded Circle');

%Now we are going to try to just filter out this expanded circle for a new

%image1 in order to test for the approximate accuracy

newim = image1;

for i = 1:1920

for j = 1:2560

if circle(i,j) == 0

binary(i,j) = 0;

end

end

end

figure

imshow(binary);

title('New Binary');

binary2 = binary;

%Now we want to make a thin circle on the perimeter and deterimne the

%percentage of white pixels in order to say if its dead or alive. For now

%we have chosen an arbitrary size of 10 pixels to search

radin = radius - 10;

circle = (row - center(2)).^2 + (col - center(1)).^2 <= radius.^2;

circle((row - center(2)).^2 + (col - center(1)).^2 <= radin.^2) = 0;

circle = mat2gray(circle);

figure

imshow(circle);

title('Thin Circle');

for i = 1:1920

for j = 1:2560

if binary2(i,j) == 0

circle(i,j) = 0;

end

end

end

figure

imshow(circle);

title('Percentage Circle?');

%Now we want to count the percentage of white pixels on the perimeter

maxpixelarea = (pi*radius^2) - (pi*radin^2);

pixelarea = sum(circle(:));

percentage = (pixelarea/(maxpixelarea))*100;

disp('The Percentage of white pixels in the region is: ');

disp(percentage);

else

disp('No Follicle Detected in this Image');

end
